# Strategies to Improve Bone Healing: Innovative Surgical Implants Meet Nano-/Micro-Topography of Bone Scaffolds

**DOI:** 10.3390/biomedicines9070746

**Published:** 2021-06-28

**Authors:** Dirk Wähnert, Johannes Greiner, Stefano Brianza, Christian Kaltschmidt, Thomas Vordemvenne, Barbara Kaltschmidt

**Affiliations:** 1 Department of Trauma and Orthopedic Surgery, Campus Bielefeld-Bethel, Burgsteig 13, Protestant Hospital of Bethel Foundation, University Hospital OWL of Bielefeld University, 33617 Bielefeld, Germany; Dirk.Waehnert@evkb.de (D.W.); Thomas.Vordemvenne@evkb.de (T.V.); 2 Forschungsverbund BioMedizin Bielefeld/OWL FBMB e.V., Maraweg 21, 33617 Bielefeld, Germany; Johannes.Greiner@uni-bielefeld.de (J.G.); C.Kaltschmidt@uni-bielefeld.de (C.K.); 3 Department of Cell Biology, Bielefeld University, Universitätsstrasse 25, 33615 Bielefeld, Germany; 4 Biomech Innovations AG, Aarbergstrasse 5, 2560 Nidau, Switzerland; Stefano.Brianza@biomech-innovations.com; 5 Molecular Neurobiology, Bielefeld University, Universitätsstrasse 25, 33615 Bielefeld, Germany

**Keywords:** fracture healing, mechanical environment, bone substitute material, nanotopography, microtopography

## Abstract

Successful fracture healing is dependent on an optimal mechanical and biological environment at the fracture site. Disturbances in fracture healing (non-union) or even critical size bone defects, where void volume is larger than the self-healing capacity of bone tissue, are great challenges for orthopedic surgeons. To address these challenges, new surgical implant concepts have been recently developed to optimize mechanical conditions. First, this review article discusses the mechanical environment on bone and fracture healing. In this context, a new implant concept, variable fixation technology, is introduced. This implant has the unique ability to change its mechanical properties from “rigid” to “dynamic” over the time of fracture healing. This leads to increased callus formation, a more homogeneous callus distribution and thus improved fracture healing. Second, recent advances in the nano- and micro-topography of bone scaffolds for guiding osteoinduction will be reviewed, particularly emphasizing the mimicry of natural bone. We summarize that an optimal scaffold should comprise micropores of 50–150 µm diameter allowing vascularization and migration of stem cells as well as nanotopographical osteoinductive cues, preferably pores of 30 nm diameter. Next to osteoinduction, such nano- and micro-topographical cues may also reduce inflammation and possess an antibacterial activity to further promote bone regeneration.

## 1. Introduction

Bone is a living organ that is subject to a continuous modeling and remodeling process throughout its life [1,2]. This process is under complex control and follows the principle first described by Wolff in 1870: form follows function [3]. In this way, the bone is able to adapt to changing circumstances (e.g., mechanical load). After an injury (fracture), bone is one of the few organs that can heal without an inferior connective tissue scar. Bone heals with the emergence of bone tissue with the original (pre-fracture) properties [1,4].

In 2019, more than 620,000 fractures of the extremities were treated as inpatients in Germany. Therefore, fractures are one of the most common causes of inpatient treatment following an accident. Nevertheless, there are complications in bone healing, and the rate of non-healing fractures is estimated to be between 1.9% and 10% of all fractures [5,6]. It is estimated that each year 100,000 fractures in the United States develop a fracture healing complication (non-union) [7]. The risk of developing fracture healing complications depends on numerous factors. Patient dependent factors include advanced age, male gender, various medical comorbidities, smoking, use of nonsteroidal anti-inflammatory drugs, metabolic diseases, and nutrient deficiencies [5,8,9]. Patient independent factors are the anatomical region of the fracture, fracture morphology, the extent of soft tissue damage, the type of surgical stabilization, and the presence of infection [6,10,11,12,13]. A further classification of the causative factors of bone healing complications distinguishes between factors resulting from inadequate mechanical stability and/or surgical therapy and those factors resulting from impaired biological activity [14]. Complications in the healing of a bone fracture have consequences for the patient. In addition to pain, physical limitations and mental stress, there are recurrent hospitalizations, multiple surgical interventions and reduced quality of life. However, complications of fracture healing also represent relevant sources of costs for society and the health care system. According to the calculations of Ekegren and coworkers, the direct costs of treating complications of bone healing in an Australian collective range from 4788 to 93,197 Australian dollars per patient [15]. In the United States, the median cost of treating a non-union after open tibial fracture is estimated at 25,556 USD [16]. In addition to non-unions, infections are a relevant complication of fracture healing. Galvani and coworkers were able to show that the direct costs in patients with infections after surgically treated tibia fracture were 80% higher than in patients without complications [17]. All of these estimates are for the direct costs of treatment; however, indirect costs represent the major portion of the total costs of treating patients with complications of fracture healing. For example, Hak and coworkers put the proportion of indirect costs between 67% and 79% for the Canadian health care system and between 82.8% and 93% for the European health care systems [7].

According to the current understanding of physiological bone healing, a coordinated interaction of molecular, physical and biomechanical factors in complex pathways is necessary [18]. In this process, the sequential stages of embryonic endochondral bone formation are repeated during fracture healing. The study of these biophysical processes of bone healing has led to the identification of a variety of signaling cues that regulate cellular activity and tissue development [19]. However, the translation of the results into clinical application is difficult, because the effect of many signals in the process of bone healing has a significant chronological and temporal component. One approach to address this problem are biomimetic drug delivery systems that allow control over the location, timing and delivery kinetics of signal molecules [19]. Furthermore, physical factors have a significant influence on bone healing. They affect the shape, function and activity of bone-related cells such as mesenchymal stem cells, osteoblast, osteoclasts and osteocytes [19]. Mechanical stimulation can positively influence osteogenic differentiation, but also mineralization [19,20]. Moreover, a mechanically optimized scaffold can induce osteogenic differentiation of mesenchymal stem cells [19] (see also Section 3.5). In clinical practice, these physical factors are applied, for example, in the procedures of focused extracorporeal shock wave therapy or pulsed electromagnetic fields [21,22].

**Figure 1:**
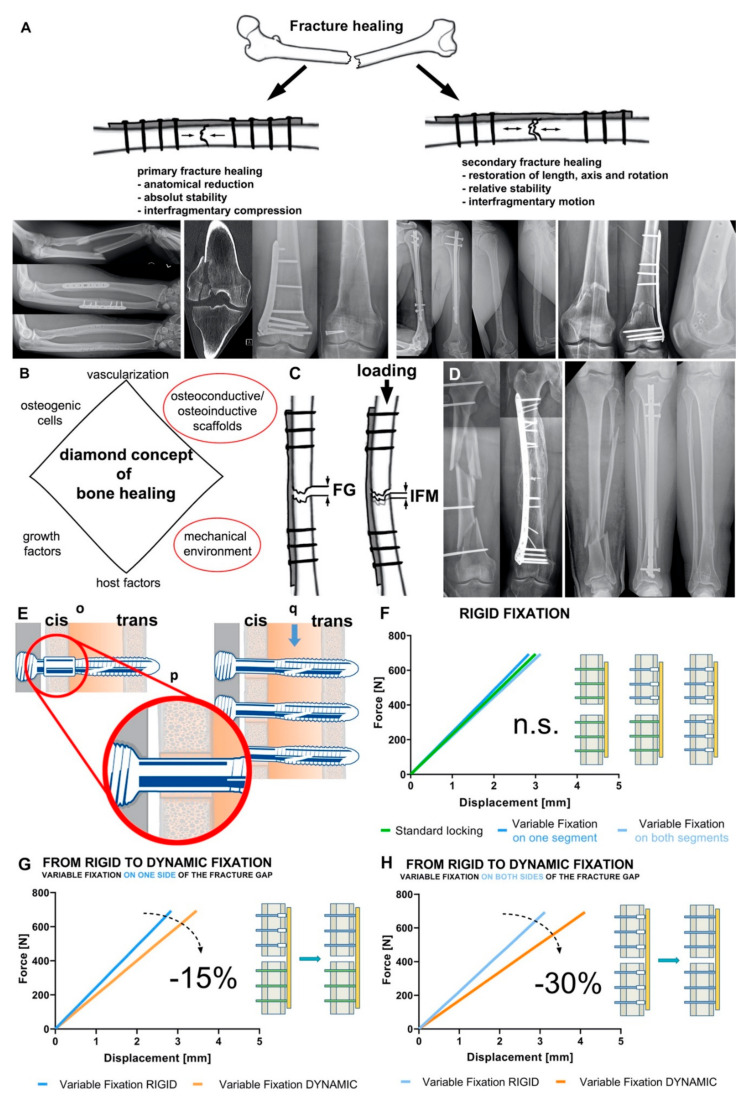
Mechanical environment is crucial for bone homeostasis and fracture healing. (**A**) Types of fracture healing. Primary fracture healing (left) requires interfragmentary compression, the x-rays showing two clinical cases: forearm fracture treated with two compression plates and healing without callus formation and intraarticular distal femur fracture treated with lag screw and plate osteosynthesis. Secondary fracture healing (right) requires interfragmentary motion, the x-rays showing two clinical cases: fracture of the humeral diaphysis treated by intramedullary nailing and healing with callus formation and supracondylar fracture of the distal femur treated by angular stable bridge plating resulting in healing via callus formation and maturation. (**B**) Diamond concept of fracture healing modified according to Giannousdis et al. [23,24]. (**C**) Interfragmentary strain is defined as the result of the inter fragmentary movement (IFM) resulting from loading divided by the fracture gap (FG) size. (**D**) Influence of bone-implant construct stiffness on fracture healing and callus amount. Complex femoral shaft fracture (left) treated with a long plate allowing interfragmentary motion and healing with a large callus. Tibial shaft fracture (right) treated with an intramedullary nail with high stiffness resulting in healing with lower callus volume. (**E**) the Variable Fixation Locking Screw (VFLS) implanted in cortical bone (o). The resorbable sleeve centers the screw in the cis-cortex hole (p) allowing all screw (q) to truly work in parallel. (**F**) When the resorbable sleeve is intact, there is no significant difference among the stiffness of construct fixed with a plate and standard locking, variable fixation or mixing the two technologies. (**G**) The transition between rigid and dynamic fixation produces a 15% decrees in construct stiffness using variable fixation on one side of the fracture gap, while the transition between rigid and dynamic fixation produces a 30% decrees in construct stiffness using variable fixation on both side of the fracture gap (**H**).

Two basic types of bone healing can be distinguished histologically into primary and secondary fracture healing (Figure 1A) [23]. Primary or direct fracture healing is rather rare and is based on a direct attempt by the cortical cells to restore the interrupted continuity in the context of remodeling. Primary healing requires direct contact of the bone fragments and so-called absolute stability with minimization of interfragmentary strains. Secondary or indirect fracture healing is the more common form of healing and is found in the vast majority of fractures. Indirect fracture healing proceeds in well-characterized phases. In the first phase (inflammation), a hematoma forms in the fracture gap immediately after bone fracture. Such hematoma is infiltrated with inflammatory cells like macrophages, granulocytes and mast cells. The secretion of mediators (e.g., cytokines, growth factors) into the fracture hematoma regulates the differentiation of mesenchymal stem cells into osteoblasts, fibroblasts and chondrocytes as well as cell infiltration and angiogenesis. In the following phase (granulation phase), the hematoma is replaced by granulation tissue mainly characterized by fibroblasts, further collagen and capillaries. This tissue is now called soft callus. This phase is also characterized by the resorption of non-perfused bone substance in the fracture gap. In the subsequent phase, the callus matures through mineralization of the extracellular matrix. The resulting woven bone organizes itself according to the mechanical stresses acting on the forming tissue and the fractured bone finally regains its mechanical competence. Lastly, during the modeling/remodeling phase, the woven bone is replaced by organized lamellar bone and the macroscopic bone shape (including the medullary cavity) is fully restored. Both forms of fetal bone formation, the intramembranous and endochondral ossification occur. In intramembranous ossification, bone is formed directly by mesenchymal stem cells differentiating into osteoblasts and secreting osteoid. Small trabeculae are formed, which fuse to form woven bone. Endochondral ossification is characterized by indirect bone formation via an intermediate stage of cartilage (callus). Lamellar bone is formed in this process [4]. The prerequisites for secondary healing are the activation of bone-forming cells, a so-called relative stability of the fracture and sufficient blood supply. Nevertheless, disturbances of bone regeneration or bone defects of critical size, which exceed the self-healing potential, occur regularly. In order to improve bone healing, or to achieve it at all, numerous biological and physical prerequisites (cells, scaffolds, factors, and micro-/nanotopography) have been scientifically investigated. In addition, mechanical stability plays a crucial role in bone healing. The biomechanical environment is essential for callus formation and maturation. It enables the formation of a sufficient, fracture-bridging and load-bearing callus.

In 2007, Giannoudis and colleagues presented their diamond concept, which summarizes the interactions of fracture healing in a plausible way (Figure 1B). The three biological factors osteogenic cells, osteoconductive/osteoinductive scaffolds and growth factors are combined with the mechanical environment at the fracture site [23]. In 2019, the concept was adapted once again and supplemented with the factors of vascularization and host factors [24].

For the purpose of this review article, we will take a closer look at two factors, mechanical environment and osteoinductive scaffolds, particularly regarding their micro- and nano-topography.

## 2. Mechanical Environment

Bone is an inhomogeneous compound tissue consisting of morphologically and mechanically different components. Whereas cortical bone has a porosity of 3% to 10%, cancellous bone has a porosity of 90% [25]. The mechanical properties differ according to localization (anatomy), direction of loading (anisotropy) and age, sex, activity, as well as bone density [26]. After a fracture, the callus is formed via granulation tissue, which in turn has different mechanical properties (Table 1).

### 2.1. Mechanical Environment on the Bone

The fundamental goals of bone healing are the restoration of mechanical resilience and strength of the bone. Both target mechanical properties, so it is not surprising that the processes involved in tissue differentiation and formation are also primarily regulated by mechanobiological feedback signals [1,34]. To be able to respond to biophysical stimuli, bone cells are coupled in a complex and dynamic way to their extracellular environment, particularly osteocytes form a dense communication network within the bone tissue and perceive the mechanical signals on the bones [25]. They regulate the survival and activity of bone-building osteoblasts and bone-resorbing osteoclasts [1,35,36]. Additionally, osteocytes “direct” fracture healing through all phases due to mechanical stimuli [1,35]. Thus, the process of mechanotransduction regulates both bone remodeling and fracture healing.

Stress, strain, fluid flow and streaming potentials are the mechanical signals induced in bone by mechanical loading. Among these, in intact bone, shear stress induced by interstitial fluid flow in the lacunar-canalicular network appears to be the most important signal for mechanotransduction [1,37]. Furthermore, it has been shown that mechanically induced deformation of the bone improves oxygen transport and thus also nutrient supply in the fracture zone [38].

At the level of the fractured bone organ, tissue deformation leads to a mechanical response, on cellular and molecular levels. Compressive strain induces the formation of cartilaginous tissue whereas tensile strains lead to the formation of fibrous connective tissue [1,39]. This might be due to the disruption of blood vessel formation by tensile and shear stresses in the early phase of fracture healing. At the cellular level, mechanical signals influence (promote or inhibit) the proliferation and differentiation of stem cells and their derivates as well as the production of extracellular matrix. For example, protein kinase B is activated by fluid shear stress, which stimulates the differentiation of mesenchymal stem cells. Furthermore, mechanical stress can directly activate Ras homolog gene family member A guanine triphosphatase in mesenchymal stem cells, leading to osteogenic differentiation [25]. At the molecular level, growth factors, cytokines and morphogens are activated in response to mechanical signals [18].

### 2.2. Mechanical Environment and Fracture Healing

It is well known that the mechanical stimulation of a fractured bone has a decisive influence on the healing response. Fracture stability can be characterized by interfragmentary strain, which is defined as the ratio between the interfragmentary movement and the fracture gap size (Figure 1C). According to the strain theory of Perren and Cordey, direct bone formation can only occur if the interfragmentary strain is smaller than the failure strain of the newly formed bone tissue [40]. Accordingly, only strains less than 2% can allow direct fracture healing [41]. This can be achieved surgically only by open anatomical reduction and absolutely stable internal fixation with interfragmentary compression (Figure 1A). Nowadays, this form of fracture healing is reserved for specific fracture patterns and indications (in e.g., joint fractures or ulnar shortening osteotomies).

The vast majority of fractures heal via the indirect or secondary route. Secondary fracture healing occurs with relative stability in the fracture area (Figure 1A). Relative stability is achieved surgically by indirect reduction and bridging internal (angular stable plate osteosynthesis, intramedullary nailing) or external fixation (external fixator) [42,43]. Even with relative stability, the right amount of interfragmentary strain must be present to achieve successful fracture healing. If the fixation is too rigid or too flexible, delayed healing or non-union will result [44,45]. This defines a loading “window of opportunity” in which fracture healing can begin and be successfully completed, which must be achieved by the trauma surgeon on an individual basis (anatomical region, fracture shape, bone quality, osteosynthesis). To modulate this “window of opportunity” in a defect situation, optimized scaffolds as discussed below (see Section 3) facilitate enhanced migration and osteogenic differentiation of stem cells, in turn improving bone healing. Clinically, fracture gap strain is determined by the loading magnitude and the stiffness of the bone-implant construct [1,46]. The size of the interfragmentary gap depends on multiple factors. The number of fragments, the location of the fracture and the type of surgical treatment (open versus closed and direct versus indirect reduction) play a decisive role. It is known that the size of the interfragmentary gap not only affects the interfragmentary strain at the fracture site, but also varies the sensitivity to different interfragmentary strains. Small gaps tolerate strains of up to 30%, whereas larger ones show delayed healing with these strains [45]. Important for the healing process is not only the amount of interfragmentary motion, but also the direction (axial, torsional, shear). Whereas axial compressive loading stimulates periosteal callus production and maturation, thus promoting fracture healing [47,48,49], shear motion leads to inhibition of callus vascularization and thus delayed fracture healing [50,51].

The amount of loading due to bone segment displacement and weight bearing directly determines the interfragmentary strain at the site of fracture. In recent years, there has been a change in mindset regarding postoperative weight bearing after extremity injuries from pre-clinical and clinical perspectives [52,53,54,55]. It has been shown that early loading of surgically treated fractures can create a stimulating strain at the fracture zone without increasing construct instability or complication rates [56,57].

### 2.3. Bone-Implant Construct Stiffness and “Dynamic” Osteosynthesis

The pioneers of osteosynthesis postulated absolutely stable and rigid fixation of fractures as the method of first choice. In their view, secondary fracture healing via callus was pathological healing that could be prevented by adequate osteosynthesis and interfragmentary compression [42]. Today we know that secondary fracture healing of limb bones is the biological pathway of bone regeneration [1]. Successful secondary fracture healing is tied to, among other things, an optimal mechanical environment deriving from construct stiffness, fracture gap motion, interfragmentary strain, and applied loading [1].

The stiffness of the bone-implant construct has a significant influence on interfragmentary motion. Whereas stiff connections between bone and implant allow little movement, less-stiff constructs are characterized by an increase in interfragmentary movement. On one hand, this stiffness is largely determined by the design and the material of the implant. On the other hand, the osteosynthesis configuration (e.g., screw configuration, double plating) significantly affects the construct stiffness. For example, in the treatment of tibial fractures, intramedullary nails have a significantly higher axial stiffness than extramedullary implants (e.g., angular stable plates, external fixator), whereas torsional stiffness and shear stress are comparable (Figure 1D) [58,59,60]. Due to this property, after implantation of an intramedullary nail, dynamization of the osteosynthesis by removal of locking bolts is often necessary to stimulate fracture healing.

For a long time, biomechanical studies have argued that high stiffness of implants or bone-implant constructs is beneficial for the treatment of fractures [61]. However, recent studies show that dynamic fixation of diaphyseal fractures improves fracture healing. In these studies, dynamic fixation was generated either by modification to the screws or to the plate [62,63,64,65,66]. All these techniques have in common that dynamic component of fixation is constant over the time of fracture healing.

However, various studies have shown that the time at which interfragmentary motion occurs also has a decisive influence on fracture healing. The phases of fracture healing (inflammation, repair with soft callus formation, callus maturation and calcification, remodeling) differ in both tissue composition, cellular and vascular activities, and most importantly, mechanical integrity [67]. The early inflammatory phase is characterized by revascularization at the fracture site, so it is reasonable to assume that increased interfragmentary motion negatively affects the formation of new vessels [1,68,69]. On the other side, stimulating interfragmentary motion by dynamic fixation during the phase of soft callus formation seems to be reasonable because the soft callus already contains chondrogenic cells [70,71] and bone precursor cells able to respond to the mechanical stimuli [72]. The result would be faster maturation of the soft callus and increased mineralization and remodeling of the hard callus [72].

In summary, the current literature indicates that there is a fairly large strain “window of opportunity” in which fracture healing occurs. However, the inflammatory phase seems to profit from a stable mechanical environment that allows differentiation of mesenchymal cells towards an osteogenic lineage. In turn, mechanical stimulation during early callus formation rather than during later stages [73,74,75] may enhance stabilization through the formation of additional cartilage and bone.

Trying to accommodate for such emerging knowledge, new dynamic osteosynthesis screws technologies were developed and introduced to the market. The MotionLoc™ screw from Zimmer^®^ (Zimmer Biomet, Winterthur, Switzerland) enables dynamic osteosynthesis through the principle of Far Cortical Locking. The screw connects only in the plate and in the cortex far from the plate. Close to the plate, the screw diameter is smaller than the drill hole and thus allowing some additional interfragmentary movement. Bottlang and coworkers were able to show in a preclinical study that dynamic osteosynthesis with the MotionLoc™ screw led to increased and more homogeneous callus formation in the ovine tibial osteotomy model. In addition, the MotionLoc™ group showed better biomechanical properties under torsional loading [62]. Another screw with dynamic properties was the DLS (Dynamic Locking Screw) from DePuy Synthes (Zuchwil, Switzerland). The screw consisted of a hollow threaded shell with a central pin with a threaded head. This design allowed a maximum displacement movement of 0.2 mm. Preclinical studies showed a significant increase in callus formation on the sheep osteotomy model compared to the standard locking screw. In addition, the specimens of the DLS group showed a significantly higher failure moment and a higher torsional stiffness [66,76]. In 2015, this screw was withdrawn from the market due to failure of the internal pins during planned implant removal after fracture healing. Both screws (MotionLoc™ and DLS) result in immediate dynamic osteosynthesis; there is no modulation of the mechanical properties over the healing time. In addition, the MotionLoc™ screw does not provide centering in the cortex near the plate, which may result in the screws not working in parallel. This results in a random fracture dynamization and adds a non-controlled variable to the clinical work performed with these devices. Furthermore, by randomly coming earlier in contact with the cis (near) cortex (see Figure 1E) one screw is at risk of carrying the load of the entire bone segment and thus at higher risk of failure.

### 2.4. Variable Fixation Technology

We are not far from properly fixing fractures, but today it is clear that one stiffness is not good for all fractures and patients [1]. Surgeons can change the construct stiffness by assembling different configurations of screws and plates. However, the properties of the devices strongly limit the options and allow reaching only discrete changes. A new locking screw technology, called variable fixation, has been developed with the intent to align osteosynthesis devices to the state-of-the-art knowledge about bone fracture healing. This technology allows surgeons to build plate and screws constructs changing their stiffness during fracture healing time.

The Variable Fixation Locking Screws (VFLS, Biomech Innovations, Nidau, Switzerland) accommodate for all emerging requirements by means of a degradable sleeve positioned in the cis (near) cortex. The sleeve acts as screw cis-cortex hole-centering tool and guarantees that all screws work in parallel and that the dynamization can safely and effectively take place (Figure 1E). Three consecutive and timely spaced events characterize the degradation of the sleeve constituent material: decrease in molecular weight, later followed by a decrease in mechanical properties and, finally, a decrease in mass with complete metabolization of the material. In a plate and variable fixation construct, this degradation process determines a period of initial stability followed by a period where, due to the decrease in mechanical properties of the material, the sleeve provides a decreasing support to the cis cortex. At the end of the degradation process the sleeve is completely resorbed and the loading is transferred from the cis to the trans cortex. The increase in the distance between the locking mechanism and the restrain point (the bending arm) determines a decrease in construct stiffness, a different trajectory of the bone fragments and mechanically stimulates the trans cortex.

Two major studies have been performed before introducing this device into clinical practice. Variable fixation technology has been tested using plates already used in clinical settings. This allowed designing studies where the variable of interest “screw technology” was the only different variable among groups and allows direct translation of the information into clinical applications.

The first is a biomechanical study where the interfragmentary stability provided by a plate and variable fixation screws was tested in a simulated fracture-gap model under compression and torsional loading [77]. The interfragmentary stability provided with intact sleeve and after its chemical dissolution was compared with that provided by the same plate and standard locking screws and with a third construct featuring the same plate and a combination of both technologies. Results showed that after implantation, constructs featuring variable fixation technology, in one or both bone segments, are as rigid as that provided by standard locking technology and the two technologies can be safely combined (Figure 1F). Furthermore, with sleeve resorption, the stiffness of the variable fixation constructs significantly decreases according to the different combinations of technologies chosen. Surgeon can obtain a progressive decrease in stiffness to about a 15% mixing standard and variable fixation technologies or 30% using variable fixation on both side of the fracture (Figure 1G,H). The recorded interfragmentary movements confirmed similar cis and trans displacements when the sleeve is intact. However, the resorption of the sleeve dynamized the gap leading to a substantial increase in displacement at the trans and, even more, at the cis cortex [77].

The second study is a preclinical investigation [78] whose results nicely validated the findings of the biomechanical study. There, callus formation was investigated on the whole bone at the fracture gap on three groups of sheep whose 3 mm tibial osteotomy has been stabilized with configurations similar to those tested in the first investigation. A progressive decrease in stiffness and a targeted change in interfragmentary displacements were shown to have an effect on the production and spatial distribution of bone callus. In the whole bone this was demonstrated by a 40% larger callus with similar mineral density in the group featuring variable fixation in one bone segment. Interestingly, in this group the bone segments implanted with variable fixation showed the trans cortex was activated and locally contributed to the stabilization of the bone fragments producing additional bone callus. Stimulated healthy cortical bone sections are the best patient-specific scaffold already in place. They feature all tissues, cells and signaling necessary to direct the repair of a defect and can potentially support the activity at the fracture gap. There, compared to standard locking technology, variable fixation had the effect of inducing larger and more homogeneously distributed callus between cortices. With a + 30% larger cis callus, variable fixation has the potential to solve the problem of insufficient callus formation under the plate using standard locking fixation [78].

The results showed also that the magnitude of variable fixation has a biological effect. Using variable fixation on both bone segments doubled the magnitude of progressive dynamization and promoted the formation of an even larger bone callus. However, this came with a slight decrease in mineral density confirming that the “window of opportunity” exists and that also the usage of variable fixation needs always to be tuned with respect to the mass of the patient, the stiffness of the chosen bone plate and size of the fracture gap [78].

In conclusion, the scientific evidence produced about this new technology suggests that variable fixation can increase the chances to bridge the fracture gap activating the entire bone organ and stimulating callus formation. Routinely used, it might increase the osteosynthesis overall success rate acting, especially on more complicated cases where a larger and homogeneously distributed amount of callus, would be making the difference. The conditions where its usage can be most beneficial for patients are currently under definition by surgeons.

## 3. Bone Scaffolds Guiding Osteoinduction—An Interplay of Micro- and Nano-Topography

Numerous bone substitute materials are applied in the field for bone replacement, thus facing the challenge of limitations in the available amounts of autologous bone, donor site morbidity and the remaining risk of immune-rejection. Such scaffolds range from bioactive ceramics and natural or synthetic polymers to composite materials (reviewed in [79]). Amongst others, natural polymers include collagen, fibrin or hyaluronic acid are applied as hydrogels based on gelatin, agarose or alginate. Synthetic polymers are often build of polyglycolic acid (PGA) or polylactic acid (PLA), while composite materials combine at least two materials as in case of poly(lactic-co-glycolic acid) (PLGA) (reviewed in [79]). Next to its mechanical properties, biocompatibility, deliverability of bioactive molecules and osteoconductivity, the osteoinductive capacity of a material is vital for proper bone healing [80]. In this regard, the distinct nano- and/or micro-topography of a given scaffold is increasingly recognized as a critical parameter for guiding osteoinduction and thus functional bone regeneration after transplantation [81]. In the present section, we will summarize recent advances in bone scaffolds guiding osteoinduction by such nano- and micro-topographical cues with a special emphasis on the mimicry of natural bone.

### 3.1. Natural Bone Architecture and Topography

**Figure 2:**
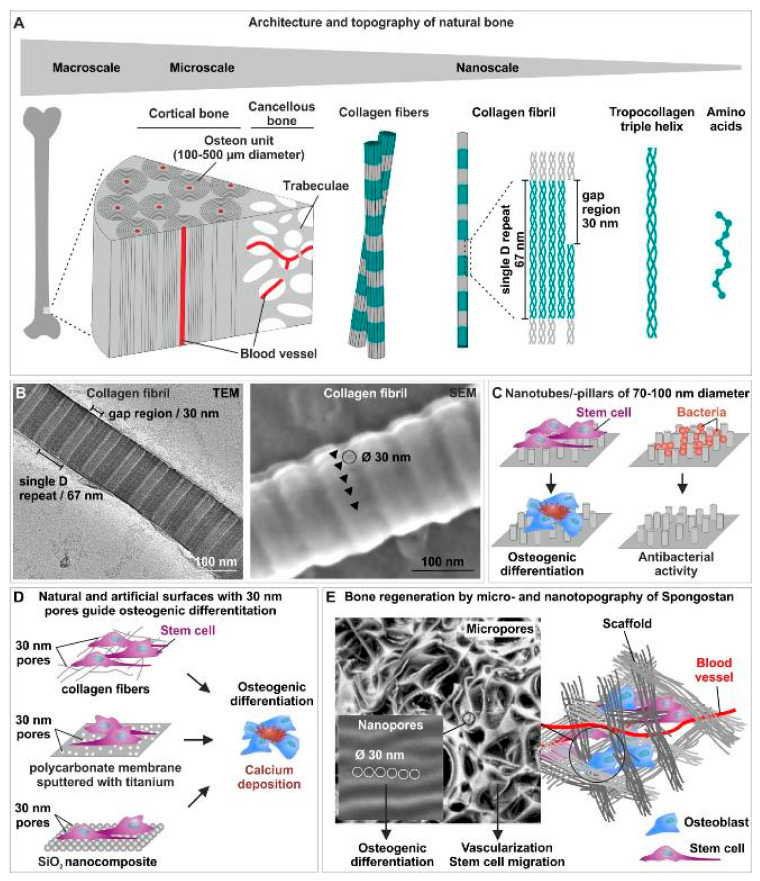
The interplay between micro- and nano-topography of scaffolds is crucial for efficient osteoinduction and bone regeneration. (**A**) Schematic view of the architecture and topography of natural bone from the macro- to the micro- and the nanoscale. (**B**) Transmission electron microscopic (TEM) and scanning electron microscopic (SEM) images of nanopores with 31.93 ± 0.97 nm diameter on the surface of collagen type I fibers [86]. (**C**) Nanotubes or nanopillars with a a diameter of 70–100 nm drive osteogenic differentiation of stem cells and possess a highly efficient antibacterial activity [89,90,92]. (**D**) 30 nm pores on collagen type I fibers [86], a polycarbonate membrane sputtered with titanium [97] or a SiO_2_ nanocomposite [86] efficiently guide osteogenic differentiation of human stem cells. (**E**) Micropores present in Spongostan allow migration of stem cells and vascularization, while osteoinductive nanopores of 30 nm diameter guide the stem cells into the osteogenic fate [87]. Parts of this figure are taken or modified from [86] (License: CC BY-NC-ND 4.0) [87] (License: Creative Common CC BY license).

The architecture and topography of natural bone are known to range from the macro- to the micro- and finally to the nanoscale. As an example, long bone can be macroscopically separated into internal cancellous bone and external compact cortical bone (Figure 2A) [79]. In the microscale, cortical bone is composed of repeating osteon units of 100–500 µm diameter, while a porous network of trabeculae forms the cancellous bone (Figure 2A). Both osteon units and trabeculae are built by mineralized collagen fibers. The microarchitecture of bone tissue allows migration of stem cells as well as vascularization and innervation (reviewed in [82]). Moving from the micro- to the nanoscale of natural bone topography, collagen fibers consist of collagen fibrils assembled by triple-helix collagen molecules, which results in a single D periodicity of 67 nm and a gap region of approximately 30 nm (Figure 2A) [83,84]. This gap region allows the embedding of hydroxyapatite (HA) crystals, making the nanotopography of collagen type I crucial for bone formation [85]. In addition, we previously reported the presence of nanopores of 31.93 ± 0.97 nm diameter on the surface of collagen type I fibers (Figure 2B), structurally closely related to the 30 nm gap region of single D repeats. We further showed these pores to efficiently induce osteogenic differentiation of adult human mesenchymal (MSCs) and neural crest-derived stem cells (NCSCs) and regeneration of calvarial critical size defects [86,87]. Thus, the micro- and nano-topography of natural bone facilitates vascularization, innervation and stem cell migration (microtopography/-architecture) as well as bone mineralization and osteoinduction of stem cells (nanotopography). These findings emphasize the therapeutic potential of biomimicking topographical cues for scaffolds.

### 3.2. Nanotopographical Cues of Scffolds Drive Osteoinduction and Possess Anti-Bacterial Activities

In accordance to the importance of their endogenous counterparts in natural bone, distinct nanotopographies of artificial or natural surfaces have been described to drive osteogenic differentiation of stem cells. In their pioneering work, Dalby and colleagues reported the fabrication of 100 nm deep pits with 120 nm diameter and a 300 nm center–center spacing on a polymethylmethacrylate (PMMA) substrate by electron beam lithography (EBL). The authors demonstrated disordered random placement of nanopits to guide osteogenic differentiation of human MSCs, while highly ordered nanopits did not drive osteogenic differentiation. Although the applied nanostructures were not specifically biomimetic, these findings provided the first evidence for nanotopographical cues directly guiding osteogenesis without biochemical stimulation [88]. Next to their order, the dimension of nanotopographical cues is a critical determinant for guiding stem cell fate decisions. Using a surface of titanium oxide nanotubes with 70–100 nm diameter, Oh and coworkers demonstrated differentiation of hMSCs into osteoblast-like cells, while the application of smaller 30 nm nanotubes only promoted cell adhesion without differentiation [89] (Figure 2C). In this line, Altuntas and colleagues recently fabricated nanopillared chitosan/gelatin films comprising pillars of 90 nm diameter and 300 nm height, which efficiently promoted differentiation of MSCs and osteoblast-like Saos-2 cells into mineralizing osteogenic cell types expressing Runt-related transcription factor 2 (RUNX2), Osteopontin (OPN) and Osteocalcin (OCN) [90] (Figure 2C). Notably, the authors also reported their nanopillared chitosan/gelatin films to possess a highly efficient antibacterial activity against Gram-positive S. aureus and Gram-negative P. aeruginosa [90], both described bone pathogens forming biofilms on implants [91] (Figure 2C). The antibacterial properties of the nanopillared chitosan/gelatin films were suggested to be based on an nearly ideal surface energy or on physical interactions, particularly resulting in a trap of bacteria between the nanopillars, in turn resulting in membrane deformation [90]. In this line, Wu and coworkers showed nanopillars on an ormostamp polymer surface with a diameter of 80 nm, a pillar density of 40 pillars μm^−2^ and a roughness of 39.1 nm to possess a high bactericidal efficiency against S. aureus [92] (Figure 2C). The nanotopography of scaffolds can thus be considered as a crucial parameter for providing antibacterial and osteoinductive properties of a bone scaffold [93]. Linking the osteoinductive nanotubic topographies to nanoporous surfaces, Park and coworkers showed TiO_2_ nanotubes with lateral spacings of 15−30 nm to direct osteogenic differentiation of MSCs. On molecular level, an extensive formation of focal contacts was observable in MSCs cultured on up to 30 nm openings, indicating an active integrin-mediated signaling in turn guiding differentiation [94]. Particularly the integrin subunits β_1_ and α_2_ are broadly described to be upregulated by topographical cues, in turn leading to increased phosphorylation and activation of focal adhesion kinase (FAK) and thus expression of osteogenic transcripts BMP, ALP, OPN, OCN and Osterix [95,96]. In line with the findings by Park and coworkers, our group previously demonstrated randomly distributed pores of 30 nm diameter on a polycarbonate membrane sputtered with titanium to be sufficient for guiding osteogenic differentiation of human NCSCs (Figure 2D). In contrast, application of a nanotopography comprising 100 nm pores or a flat titanium surface was not sufficient to drive osteogenic differentiation. Notably, only NCSCs cultivated on 30 nm pores revealed a highly increased level of FAK-phosphorylation accompanied by elevated expression of the Integrin subunits β_1_ and α_2_, resulting in osteogenic differentiation [97]. Differentiation of NCSCs and MSCs was likewise achieved using a SiO_2_ nanocomposite harboring 30 nm pores, indicated by the presence of Alizarin Red S-positive calcium deposits [86] (Figure 2D). From a biomimetic perspective, these artificial porous surfaces closely resemble the 30 nm nanopores present on collagen type I fibers as discussed above [86] (Figure 2A,B,D).

Applying a further critical parameter for early bone healing, implantation of screw-shaped implants with nanopores ranging from 16–28 nm was recently reported to result in rat tibiae regeneration even under micromotion (up to two loadings with a force of 1.5 N per day) [98]. Interestingly, Marino and colleagues reported the successful production of a trabecula-like structure resembling the typical microenvironment of trabecular bone cells by two-photon polymerization. The authors showed osteogenic differentiation of SaOS-2 bone-like cells upon culture on the produced structures, resulting in increased hydroxyapatite production [99]. Notably, the volume of nanopores also seems to influence osteoinductive properties of nanotopographical scaffolds. Hayashi and Ishikawa recently reported the production of three different honeycomb scaffolds from carbonate apatite and bone mineral all harboring nanopores of nearly equal sizes (all ranging from 20–200 nm), but with different volumes of 0.18, 0.15 and 0.07 cm^3^ g^−1^. While transplantation of the scaffold harboring pores of 0.07 cm^3^ g^−1^ volume into rabbit femur critical-sized bone defects revealed promoted bone neoformation, nanopore volumes of ≥0.18 cm^3^ g^−1^ resulted in highly increased osteoclastogenesis leading to resorption of the newly formed bone. Notably, transplantation of the scaffold with nanopores of 0.15 cm^3^ g^−1^ volume resulted in a balance between osteoclastogenesis and osteogenesis required for optimal bone regeneration [100]. Notably, all scaffolds applied by Hayashi and Ishikawa comprised not only nanopores, but also micropores of 98.6 μm diameter, indicating the additional relevance of micropores for bone substitute scaffolds [100].

### 3.3. Linking the Nano- and the Microscale: Bone Scaffolds Combining an Optimal Microtopography for Vascularization and Stem Cell Migration with Nanotopographical Osteoinductive Cues

From a microscale perspective, bone scaffold topographies should allow vascularization, innervation and migration of stem cells or osteoprogenitors, proper cell arrangement and finally bone growth [79,82,101]. These requirements are broadly accepted to be optimally met by scaffolds harboring micropores with 50–150 µm diameter [79,101,102]. In terms of osteoconductivity, optimal sizes of micropores are discussed to range around 100 µm or even >100 µm [103,104]. Accordingly, a channel-like pore architecture with micropores of 89 ± 15 µm diameter was shown to be sufficient for regeneration of a rat femur critical-size model by Petersen and colleagues [105]. Extending the perspective on sole micro- or nanotopographical cues, scaffolds based on the endogenous topography of bone in the micro- and the nanoscale are increasingly recognized as potential bone substitute materials. In the following, we will particularly shed light on scaffolds fabricated from mineralized collagen or sole collagen itself as most direct approaches to biomimicking the composition of natural bone.

In terms of mineralized collagen, Liu and colleagues reported the fabrication of a biomimetic scaffold based on hierarchical intrafibrillar mineralized collagen with a nanotopography mimicking endogenous bone [106,107]. Hierarchical mineralized collagen fibrils building the basis of the scaffold were produced by a bottom-up approach including the formation of collagen fibrils from tropo-collagen molecules followed by apatite crystallization [108]. Like their endogenous counterparts, mineralized collagen fibrils exhibited a periodicity of 66 ± 1.3 nm with respective gap regions serving for deposition of apatite crystals [106]. Sole mineralized collagen fibrils successfully guided osteogenic differentiation of rat bone marrow stem cells (BMSCs) and human periodontal ligament stem cells by their nanotopography, indicated by alkaline phosphatase (ALP) activity and the presence of osteogenic marker proteins [106,109]. Assembly of mineralized collagen fibrils resulted in a highly porous scaffold with micropores of 155.88 ± 30.91 μm diameter and the nanotopographical cues discussed above, allowing regeneration of critical-size mandibular defects after co-transplantation with BMSCs in vivo [106]. Three years later, the group around Liu reported regeneration of rat mandible defects even after transplantation of mineralized collagen scaffold without pre-loading with stem cells or cytokines. On cellular level, the hierarchical nanointerface of the scaffolds led to recruitment and differentiation of endogenous MSCs accompanied by increased polarization of M2 macrophages, which was suggested to alleviate inflammation of the lesion and promote MSC differentiation [107].

Next to mineralized collagen, the micro- and nano-topography of sole unmineralized collagen scaffolds is highly promising for inducing bone regeneration. As discussed above, Petersen and coworkers demonstrated healing of a rat femur critical-size model via endochondral ossification after implantation of an engineered porcine collagen scaffold. The respective scaffold comprised a channel-like pore architecture with micropores of 89 ± 15 µm diameter, allowing recruitment of adult osteochondral progenitor cells as well as vascularization, in turn resulting in an organized endochondral ossification process [105]. Extending these findings towards intramembranous ossification, we recently reported the presence of micropores with 60.66 ± 24.48 µm diameter accompanied by nanopores of 32.97 ± 1.41 nm diameter in pre-wetted Spongostan. Spongostan is a clinically-approved collagen sponge applied in a broad range of clinical settings as a hemostatic agent being degradable, biocompatible, inexpensive and non-allergenic. We observed the micro- and nano-topographical features identified in pre-wetted Spongostan to efficiently guide osteogenic differentiation of human stem cells and regeneration of a critical-size calvarial rat bone defect. On the contrary, masking the nano- and micro-topography of Spongostan by applying nanoparticles of 146 nm diameter in thick 3D layers completely inhibited bone regeneration in vivo. Our findings thus revealed that the microporous architecture of Spongostan allowed migration of stem cells into the scaffold, while the nanotopographical cues induced their osteogenic differentiation [87] (Figure 2E). These findings are in line with our previous observations showing an osteoinduction of human stem cells solely by 30 nm nanopores present on collagen type I fibers [86] (Figure 2D). Very recently, our group directly compared the regenerative effects of Spongostan and its distinct micro- and nano-topography with the clinically applied organic bone substitute materials NanoBone and Actifuse [110]. NanoBone is composed of nanocrystalline hydroxyapatite embedded in nanostructured silica gel with 10–20 nm nanopores [111], while the bone substitute material Actifuse consists of calcium phosphate with silicate substitution harboring micropores of 200–500 µm. Although transplantation of all three substitute materials into rat calvarial critical size defects resulted in increased bone volume compared to control, application of Spongostan led to the best bone regeneration in comparison to NanoBone and Actifuse [110]. These observations further emphasize collagen-based scaffolds with nano- and micro-topographical cues of native bone to be highly promising for improving bone regeneration.

### 3.4. Bone Scaffolds Combining Topographical Cues with Stem Cell-Loading

For treatment of large bone defects, osteoporosis and aged individuals, normal scaffolds might be insufficient for proper regeneration. In such case, it might be necessary to load scaffolds with stem cells (MSCs, NCSCs) to improve bone regeneration. As discussed above, co-transplantation of mineralized collagen scaffolds with BMSCs was shown to result in regeneration of mandibular critical-size defects in vivo [106]. Oryan and coworkers reported the production of gelatin/nano-hydroxyapatite scaffolds with bioactive glass resulting in micropores of 323 μm mean pore size, while the authors did not investigate the scaffold nanotopography. Transplantation of the scaffolds loaded with MSCs into rat critical size radial bone defects showed elevated bone formation compared to the cell-free scaffolds after 4 and 12 weeks [112]. Accordingly, Tortelli and coworkers showed MSC-loaded ceramic implants to result in endochondral ossification in mice accompanied by elevated vascularization of the scaffold [113]. As an outline, nano-fibrous scaffolds produced by electrospinning, which mimic natural bone architecture and morphology, are increasingly applied in combination with MSCs to promote bone healing (reviewed in [114]).

### 3.5. Dynamizing Scaffold Design: Magnetic Scaffolds for Bone Regeneration

In addition to their nano- and micro-topography, managing to effectively dynamize bone scaffolds with a phase-bonded mechanobiological stimulus might be the key for a further step in fracture healing. Next to the concept of variable fixation technology introduced in Section 2.4, the application of magnetic scaffolds is highly promising for dynamizing implant technology. While static magnetic fields have been reported to be solely sufficient for promoting bone formation, incorporation of magnetic nanoparticles into bone scaffolds offers even greater possibilities for bone repair (reviewed in [115]). In this line, magnetic nanoparticles were shown to induce osteogenic differentiation of MSCs upon internalization under a static magnetic field [116]. Combined with stem cell-loaded scaffolds (Section 3.4), such magnetic nanoparticles were shown to strongly increase bone regeneration. On molecular level, enhancements in bone repair were discussed to involve integrin, BMP and NF-κB-signaling (reviewed in [117]). Notably, this promising approach can be directly linked to the application of collagen scaffolds discussed above (Section 3.3). Here, Marcacci’s group introduced in 2013 a bone scaffold system including the transplantation of permanent magnets close to collagen/hydroxyapatite scaffolds magnetized by magnetic nanoparticles [118]. Three years later, the same group reported the successful implantation of such permanent magnets together with the magnetized collagen/hydroxyapatite scaffolds into rabbit femoral condyle defects. The authors observed superior bone formation including the presence of highly-interconnected trabeculae 12 weeks post-surgery compared to controls [119]. Despite these promising findings, the scaffold charge [120], field gradient as well as forces exerted on magnetic nanocarriers in a static magnetic field have to be critically considered for moving such approaches from bench to bedside (reviewed in [121]). From a therapeutic perspective, magnetic implants nevertheless possess a great potential for fracture healing and may be further linked to scaffolds harboring a defined micro- and nano-topography optimal for bone growth as discussed above. Interestingly, magnetic scaffolds may even offer new possibilities for the treatment of bone tumors such as osteosarcoma. Here, surgery and/or chemotherapy is followed by local hyperthermia treatment increasing the temperature of cancer target tissue up to 43 °C, which is facilitated by the implantation of magnetic biomaterials utilized as thermoseeds. Such magnetic scaffolds allow the application of local hyperthermia even post-surgery, thus effectively inhibiting tumor recurrence [122,123].

## 4. Conclusions

In summary, the findings reviewed here emphasize the need to critically consider both the mechanical environment as well as the micro- and nano-topography of implants during fracture healing. Regarding the mechanical environment, we discuss variable fixation technology as a new implant concept offering the ability to change its mechanical properties. This advancement was shown to result in increased and more homogeneous callus formation and thus improved fracture healing. Linking this novel implant technology to recent improvements in implant architectures and topography, we further highlight the need to critically consider both micro- and nano-topographical cues of scaffolds for efficient guiding of bone regeneration in the patient. In particular, pores in the microscale (50–150 µm diameter) are required to allow migration of stem cells and their recruitment to the site of regeneration as well as vascularization and innervation of the scaffold and the newly formed bone (Figure 2E). After homing of stem cells into the scaffold, osteoinductive nanotopographical cues (preferably pores of 30 nm diameter) are needed to drive them into the osteogenic fate (Figure 2E). Interestingly, in a computational approach, Moreo and coworkers tried to model main biological interactions at the bone implant surface by reaction-diffusion equations, suggesting the possibility of an in silico simulation of implant geometry and topography in the future [103]. In this line, Mirulla and colleagues very recently systematically reviewed the progress in periprosthetic bone remodeling through finite element (FE) simulation [104]. The authors conclude an increasing interest in the bone remodeling FE analysis, while more than half of the analyzed studies utilized the adaptive bone-remodeling theory proposed by Huiskes and coworkers [105], which mainly depends on the rigidity and the implant bonding characteristics [104]. These studies suggest a future possibility to quantitatively improve scaffold design in silico by further incorporating the here discussed advances regarding scaffold nano- and micro-topography as well as new implant concepts. From a cell biological view, loading of scaffolds with stem cells may further improve their capacity for bone regeneration for treating large bone defects, osteoporosis and aged individuals. As a future perspective, the combination of such micro- and nano-topographical cues is increasingly suggested to reduce inflammation via macrophage polarization and possess an antibacterial activity, thus further contributing to bone healing. Finally, managing to effectively dynamize such promising bone scaffolds with a phase-bonded mechanobiological stimulus might be the key for a further step in fracture healing. In this line, the linkage between new implant concepts like variable fixation technology with a defined micro- and nano-topography of the implant as discussed above may result in an improved clinical bone healing in patients.

## Figures and Tables

**Table 1:** Mechanical properties of different types of bone tissue determiend in biomechanical studies.

	Trabecular Bone	Cortical Bone	Callus	Granulation Tissue
Peak load (MPa)	2.2–6.8 [27,28,29]	109–205 [30,31]	5.3 [32]	-
E-modulus (MPa)	74–900 [27,28,29]	10,460–17,100 [30,31]	98 [32]	-
Indentation modulus (MPa)	-	132 [33]	2.89 [33]	0.99 [33]

## Data Availability

The data presented in this study are available on request from the corresponding author.
